# A high-sensitivity rotatable 3D displacement sensor

**DOI:** 10.1038/s41598-023-32178-3

**Published:** 2023-03-29

**Authors:** Jianxian Cai, Tao Jiang, Zhitao Gao, Yan Shi

**Affiliations:** 1 Institute of Disaster Prevention, School of Electronic Science and Control Engineering, Sanhe, 065201 Hebei China; 2 Hebei Key Laboratory of Earthquake Disaster Instrumentation and Monitoring Technology, Sanhe, 065201 Hebei China

**Keywords:** Natural hazards, Engineering

## Abstract

Aiming at the problems of low sensitivity and low accuracy caused by the displacement transfer mechanism of three displacement sensors used simultaneously in the 3D displacement monitoring of seismic isolation bearings, the paper has proposed a high-sensitivity rotatable 3D displacement sensor. The sensor adds through holes on the surface of the equal-strength cantilever beam to form a cross beam, which increases the bending strain on the beam surface to improve the sensitivity. By adding a gyroscope and a mechanical rotation structure, a single sensor can measure the 3D displacement at the same time, reducing the adverse effects displacement transmission mechanism on the accuracy of the measurement. ANSYS software was used to simulate and optimize the parameters of the size of through-hole of the sensor beam to determine the appropriate size and location of the through-hole. Finally, the sensor was developed and its static characteristics and displacement measurement performance in static and dynamic 3D space were tested based on the simulation results. The test results have shown that the sensor has a sensitivity of 16.29 mV/mm and an accuracy of 0.9% in the range of 0–160 mm. Its static and dynamic 3D spatial displacement measurement errors are less than 2 mm, which can meet the accuracy requirements of 3D displacement measurement and sensitivity for structural health monitoring of seismic isolation bearings.

## Introduction

In recent years, earthquake disasters have occurred frequently. The structural damage to houses occurring during earthquakes has brought unbearable losses to people^1^. Traditional earthquake proofing techniques are far from expectations in earthquakes^2^. To fundamentally reduce the seismic energy input to the structure, engineers have shifted their focus from traditional "seismic resistance" to "seismic isolation"^3^. The seismic isolation structure is generally a flexible seismic isolation layer between the foundation and the upper structure, so that the foundation is to some extent disconnected from the upper structure^4^. In this way, the transmission of seismic energy to the upper structure can be isolated, basic self-oscillation frequency of the upper structure can be reduced, thus reducing the impact of seismic action on the upper structure^5^. By taking the seismic isolation measures, the self-oscillation frequency of the upper structure can generally be reduced from 1–6 Hz to 0.2–0.5 Hz, which can significantly reduce the impact of seismic forces and effectively resist the direct and secondary disasters generated by earthquakes^6^. The seismic isolation bearing is the key component of the seismic isolation structure system. Due to the complex self-load and environmental load effects during the construction and use, the structural system damage will inevitably accumulate, resulting in a decline in the ability of seismic isolation bearing to resist natural disasters^7^. Not only will it affect the normal use of the support structure, but it will also bring certain safety hazards to the building and increase the risk of casualties and economic losses caused by the earthquake^8^. Therefore, the health status of seismic isolation bearings in volatile environments has become the focus of many scholars^9^. The traditional evaluation method of seismic isolation bearings is manual evaluation, that is, manually dismantling the seismic isolation bearings to be tested, and then evaluating their health status based on appearance, load test and so on^10^. However, actually there are a large number of seismic isolation bearings in large-scale engineering buildings and the installation environment is complex^11^. Manual evaluation is not only time-consuming, laborious, but also costly^12^. With the rapid development of sensor technology, information collection technology and test analysis technology, the real-time and continuous health monitoring system of seismic isolation bearings has been widely used in bridges, high-rise buildings, water conservancy and other engineering fields^13^.

In recent years, displacement sensors have been extensively and intensively studied at home and abroad. Niu et al.^14^ proposed a resistance strain type displacement sensor, which uses strain gauges to convert the bending deformation of tool steel into displacement values. The measurement range of this structure is 0–500 mm, but the sensitivity is only 0.098. Lu et al.^15^ proposed the FBG displacement sensor based on the elliptical amplification structure, which uses the elliptical displacement amplification mechanism to improve the sensitivity, but the sensitivity within the measurement range of 0–100 mm is only 6.1 pm/mm. Li et al.^16^ proposed a spring-embedded FBG displacement sensor, which improved the sensitivity of the sensor by indirectly pasting bare optical fiber and spring. It has a good linear response within the measurement range of 0–50 mm, and the sensor sensitivity is 23.96 pm/mm, but the overall accuracy is only about 4.94%. Jiao et al.^17^ proposed a three-dimensional spatial displacement measurement system based on the three-ball intersection positioning principle, using a fixture to fix three wire displacement sensors in pairs vertically to the steel pipe support, and the three-dimensional spatial displacement value can be obtained through spatial coordinate decomposition. The static three-dimensional spatial displacement measurement error of the system is less than 2 mm within the 0–100 mm measurement range. When using sensors to monitor the health of seismic isolation bearings, the degradation or damage of building structures can be detected without manual intervention, but there are still some limitations, including: (1) When designing the displacement sensor, the sensitivity decreases with the increase of the measuring range. When measuring in the middle and high range, the sensitivity of the sensor is already low; (2) The accuracy of health assessment results of seismic isolation bearings depends on the performance of sensors to a great extent. When installing displacement sensors with traditional methods, they are easy to be damaged when the compound displacement of the seismic isolation bearing occurs, which leads to its poor applicability; (3) In the actual monitoring process of the building, the deformation and displacement of the seismic isolation bearings is a kind of compound motion, which includes three displacement components in X, Y and Z. When measuring the displacement of a single isolation bearing, three displacement sensors should be used at the same time. However, to overcome the interference of displacement in other directions, a displacement transmission mechanism will be added to the fixed end of each sensor. In this way, it will cause the problem of excessive multi-axis accumulated error and reduce the accuracy of measurement.

Therefore, to solve the problems of low sensitivity in middle and high range measurement and low measurement accuracy caused by the deviation of displacement transfer mechanism, a high- sensitivity rotatable 3D displacement sensor was proposed in this paper. In the designed displacement sensor, a cross beam was formed by adding through-holes on the surface of the beam, and the strain gauge was pasted on the center line of the cross beam, close to the upper and lower surfaces of the fixed end. In this way, the sensitivity of the sensor was improved, and the influence of temperature on the measurement accuracy was reduced; The rotary design was used to simultaneously measure the 3D displacement of the seismic isolation bearing, which improved the measurement accuracy of the sensor. By using ANSYS^18^, the static simulation and size optimization of the designed sensor were carried out, and the displacement sensor was produced according to the simulation results. By using TD8411 rotating platform surface magnetic distribution tester and 3D six-degree-of-freedom electromagnetic vibration table, a 3D displacement test system of sensor was built, and the sensitivity and accuracy of the system were tested and analyzed.

## Structural design and measuring principle of the sensor

### Structural design of the sensor

**Figure 1:**
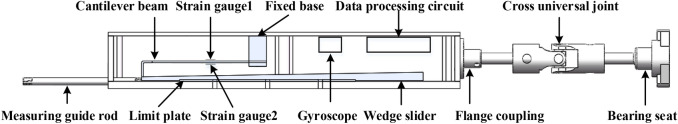
Schematic diagram of the overall structure of the sensor (The figure was generated by Microsoft Visio Professional 2016 (https://www.microsoft.com/zh-CN/download/details.aspx?id=51188)).

The overall structure of the high-sensitivity rotatable 3D displacement sensor is shown in Fig. 1. The main components include measuring guide rod, cantilever beam, limit plate, fixed base, gyroscope, data acquisition circuit, wedge slider, flange coupling, cross universal joint and bearing seat, etc. One end of the measuring guide rod was fixed with the wedge-shaped slider through threads, and the other end passed through the shell, in this way the external displacement can be transmitted to the wedge-shaped slider. The free end of the beam was always in contact with the surface of the wedge slider. The bottom of the wedge-shaped slider was installed in the chute of the bottom plate. In order to avoiding the vertical displacement of the slider to affect the measurement results, a wedge-shaped slider limit plate was fixed above the chute, so that the slider could only move along the left and right direction of the chute. Use epoxy glue to paste the strain gauges on the surfaces of the cross beam with equal strength, the upper and lower surfaces which the centerline of the cross beam near the fixed end. Strain gauge 1 was pasted on the upper surface and strain gauge 2 was pasted on the lower surface. The strain gauge and gyroscope were connected by the data acquisition circuit. The displacement and angle data were obtained in real time by the software of upper computer. After decomposition calculation of related trigonometric functions, the 3D displacement value of seismic isolation bearings was further obtained. The outer part of the sensor was connected with the cross universal joint and the bearing seat through the flange coupling, so that the sensor could rotate freely. This is convenient for measuring the yaw angle and pitch angle generated when the seismic isolation bearing drives the sensor to move.

### Theoretical analysis of the sensor and principle of 3D displacement measurement

#### Theoretical analysis of the sensor

**Figure 2:**
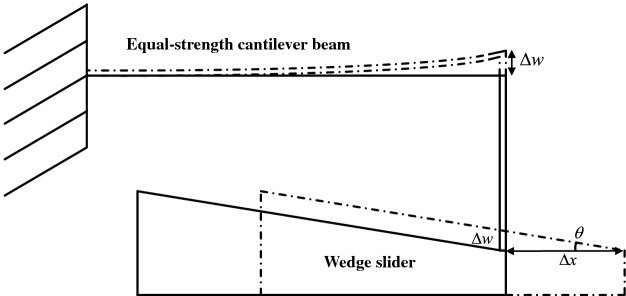
Geometric diagram inside the sensor (The figure was generated by Microsoft Visio Professional 2016 (https://www.microsoft.com/zh-CN/download/details.aspx?id=51188)).

As shown in Fig. 2, when the displacement of the sensor changes by $\Delta x$, the measuring guide rod drives the wedge slider to move by $\Delta x$, the vertical deflection of the free end of the cantilever beam is $\Delta w$, and the variation of its surface strain is $\Delta \varepsilon$. Therefore, the resistance value of the strain gauge attached to the surface of the cantilever beam changes, and the change amount is $\Delta R$.

It can be obtained from the geometric conditions:

1 $$\begin{array}{*{20}c} {\Delta w = \Delta x \cdot \tan \theta } \\ \end{array}$$

In Eq. (1), $\theta$ is the inclination angle of the inclined plane of the wedge slider.

Ignoring the influence of dead weight of the equal-strength cantilever beam, and according to the principle of material mechanics, the relationship between strain $\varepsilon$ on beam surface and vertical deflection $w$ is as follows:

2 $$\begin{array}{*{20}c} {\varepsilon = \frac{hw}{{L^{2} }}} \\ \end{array}$$

In Eq. (2), $L$ is the length of the equal-strength cantilever beam, and $h$ is the thickness of the cantilever beam.

When Eq. (1) is substituted into Eq. (2), the variation of the surface strain of the equal-strength cantilever beam can be obtained:

3 $$\begin{array}{*{20}c} {\Delta \varepsilon = \frac{ h\tan \theta }{{L^{2} }} \cdot \Delta x} \\ \end{array}$$

According to the characteristics of strain gauge itself:

4 $$\begin{array}{*{20}c} {\frac{\Delta R}{R} = k \cdot \Delta \varepsilon } \\ \end{array}$$

In Eq. (4), $\Delta R$ is the change of strain gauge resistance, R is the resistance of the strain gauge, and k is the sensitivity coefficient of the strain gauge.

**Figure 3:**
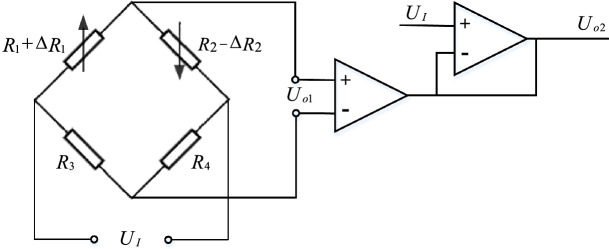
Diagram of differential bridge amplification circuit (The figure was generated by Microsoft Visio Professional 2016 (https://www.microsoft.com/zh-CN/download/details.aspx?id=51188)).

As a passive sensor that converts structural strain into resistance change, the resistance change of strain gauge can be further converted into voltage or current change by bridge circuit. Sinced the cantilever beam and the strain gauge are in the same plane, the deformation produced by the cantilever beam can be approximately regarded as the deformation of the strain gauge. When the differential bridge shown in Fig. 3 is used for detection, the output voltage can be further deduced from Eq. (4):

5 $$\begin{array}{*{20}c} {U_{o} = \left( {\frac{{R_{1} + \Delta R_{1} }}{{R_{1} + \Delta R_{1} + R_{2} - \Delta R_{2} }} - \frac{{R_{3} }}{{R_{3} + R_{4} }}} \right) \cdot U_{I} } \\ \end{array}$$

In Eq. (5), ${R}_{1}$ and ${R}_{2}$ are inductive strain gauges, ${R}_{3}$ and ${R}_{4}$ are constant resistors, ${U}_{I}$ is the input voltage, and ${U}_{o}$ is the output voltage.

When the same resistance strain gauges are connected to the adjacent arms of the bridge, that is, when ${R}_{1}={R}_{2}$ and ${R}_{3}={R}_{4}$, ${\Delta R}_{1}={\Delta R}_{2}$, and formula (5) can be simplified as:

6 $$\begin{array}{*{20}c} {U_{o} = \frac{{U_{I} }}{2} \cdot \frac{{\Delta R_{1} }}{{R_{1} }}} \\ \end{array}$$

By substituting Eq. (4) into Eq. (6):

7 $$\begin{array}{*{20}c} {U_{o} = \frac{{U_{I} k}}{2} \cdot \Delta \varepsilon } \\ \end{array}$$

By substituting Eq. (3) into Eq. (7), the relationship between the measured displacement $x$ and the output voltage ${U}_{o}$ of the differential bridge circuit can be obtained as follows:

8 $$\begin{array}{*{20}c} {\Delta x = \frac{{2L^{2} \Delta U_{o} }}{{U_{I} kh\tan \theta }}} \\ \end{array}$$

#### Principle of 3D displacement measurement

**Figure 4:**
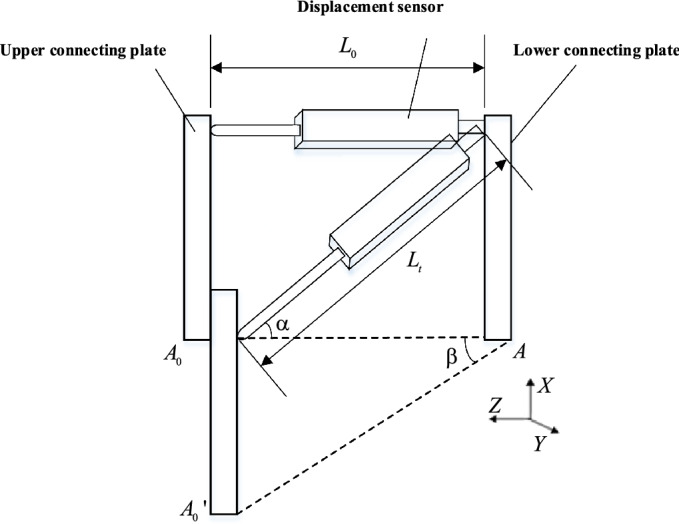
Schematic diagram of three-dimensional displacement measurement of isolation bearing (The figure was generated by Microsoft Visio Professional 2016 (https://www.microsoft.com/zh-CN/download/details.aspx?id=51188)).

To measure the displacement of the seismic isolation bearing in X, Y and Z directions simultaneously, the sensor was fixed at the position $A-{A}_{0}$ of the upper and lower connecting plate of the seismic isolation bearing, as shown in Fig. 4.

The initial stretching length ${L}_{0}$ of the displacement sensor can be calculated from Eq. (8). Gyroscope was used to collect the initial horizontal angle of the sensor, i.e. the pitch angle ${\alpha }_{0}$, and the lateral angle, i.e. the yaw angle ${\beta }_{0}$. Through the trigonometric function formula, the initial displacement values of X, Y and Z directions at the current time can be calculated as follows:

9 $$\begin{array}{*{20}c} {X_{0} = L_{0} \sin \alpha_{0} } \\ \end{array}$$

10 $$\begin{array}{*{20}c} {Y_{0} = L_{0} \cos \alpha_{0} \sin \beta_{0} } \\ \end{array}$$

11 $$\begin{array}{*{20}c} {Z_{0} = L_{0} \cos \alpha_{0} \cos \beta_{0} } \\ \end{array}$$

When the seismic isolation bearing was deformed and displaced by vibration, the position of the upper and lower connecting plate changes from $A-{A}_{0}$ to $A-{A}_{0}^{^{\prime}}$. At this time, the displacement sensor stretched and rotated with the movement of the seismic isolation bearing. The stretching length is ${L}_{t}$, and the rotation angle (pitch angle) and yaw angle become ${\alpha }_{t}$ and ${\beta }_{t}$, respectively. The displacement values in X, Y and Z directions at the current time can be calculated as follows:

12 $$\begin{array}{*{20}c} {X_{t} = L_{t} \sin \alpha_{t} } \\ \end{array}$$

13 $$\begin{array}{*{20}c} {Y_{t} = L_{t} \cos \alpha_{t} \sin \beta_{t} } \\ \end{array}$$

14 $$\begin{array}{*{20}c} {Z_{t} = L_{t} \cos \alpha_{t} \cos \beta_{t} } \\ \end{array}$$

Furthermore, the 3D displacement values of the seismic isolation bearing can be obtained as follows:

15 $$\begin{array}{*{20}c} {X = L_{t} \sin \alpha_{t} - L_{0} \sin \alpha_{0} } \\ \end{array}$$

16 $$\begin{array}{*{20}c} {Y = L_{t} \cos \alpha_{t} \sin \beta_{t} - L_{0} \cos \alpha_{0} \sin \beta_{0} } \\ \end{array}$$

17 $$\begin{array}{*{20}c} {Z = L_{t} \cos \alpha_{t} \cos \beta_{t} - L_{0} \cos \alpha_{0} \cos \beta_{0} } \\ \end{array}$$

Based on the above measurement method, the tensile displacement, pitching angle and yaw angle were measured by a rotatable displacement sensor. Through the decomposition calculation of trigonometric functions, the displacement values in X, Y and Z directions can be obtained, thus realizing the 3D displacement measurement of the seismic isolation bearing.

### Design and parameter analysis of equal -strength cantilever beam

**Figure 5:**
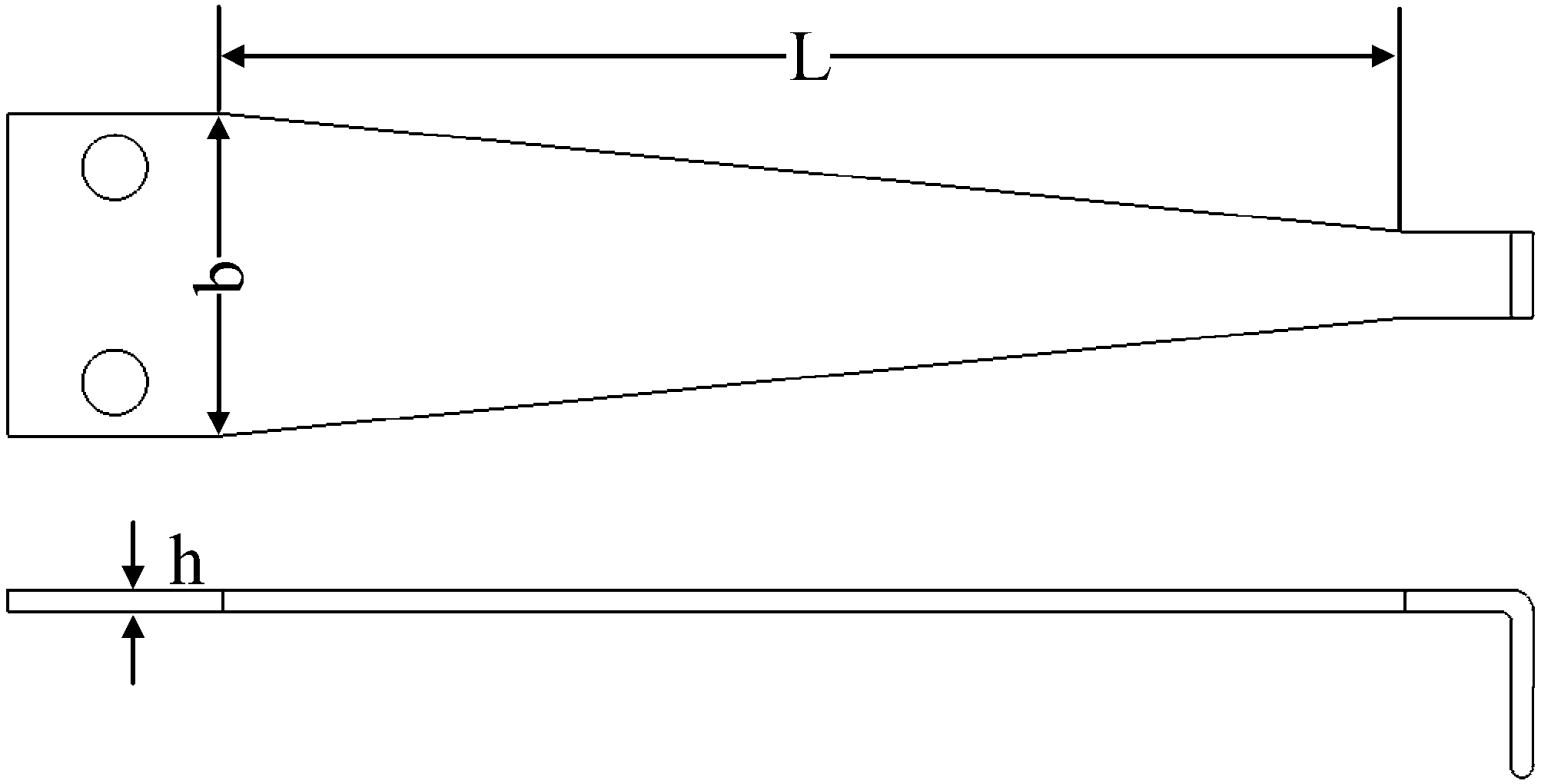
Structural schematic diagram of equal-strength cantilever beam (The figure was generated by Solidworks 2016 × 64 (https://www.solidworks.com/).

**Figure 6:**
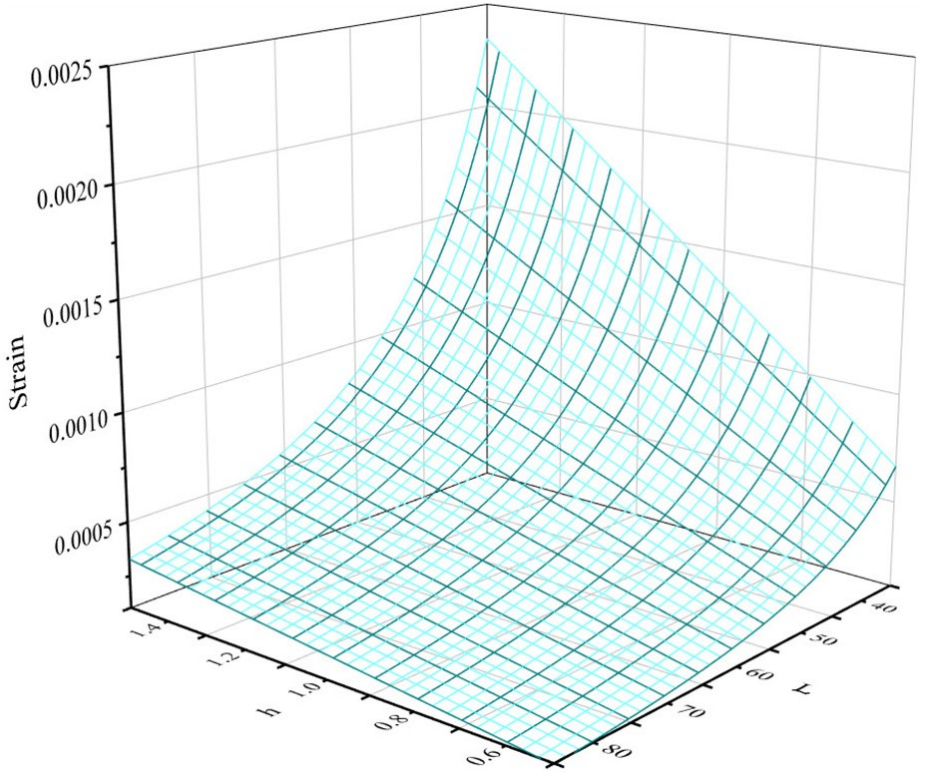
3D diagram of the relationship between the size of cantilever beam and strain (The figure was generated by Origin 2016 × 64 (https://www.originlab.com/2016)).

As the core component of displacement sensor, the size and structure of cantilever beam with equal-strength directly determines the sensitivity of the sensor. To obtain higher sensitivity, the size and structure of cantilever beam were analyzed. The cantilever structure is shown in Fig. 5, and its dimensions are: cantilever beam length $L$, thickness $h$ and beam width $b$. From Eqs. (7) and (8), it can be seen that when the input bridge voltage and the inclination angle of the chute are fixed, the sensitivity of the sensor is only related to the strain in the strain gauge area on the surface of the cantilever beam, and the strain produced on the surface of the cantilever beam is determined by its length $L$ and thickness $h$. Therefore, to obtain higher sensor sensitivity, it is necessary to design the size of cantilever beam reasonably, so that larger strain can be produced on its surface. By using Solidworks^19^ to model cantilever beams with different sizes and ANSYS to carry out static simulation analysis, this paper has investigated the relationship between the length $L$ and thickness $h$ of the cantilever beam and the surface strain of the cantilever beam, under 2.5 mm of the vertical deflection of cantilever beam. The simulation results are shown in Fig. 6.

**Figure 7:**
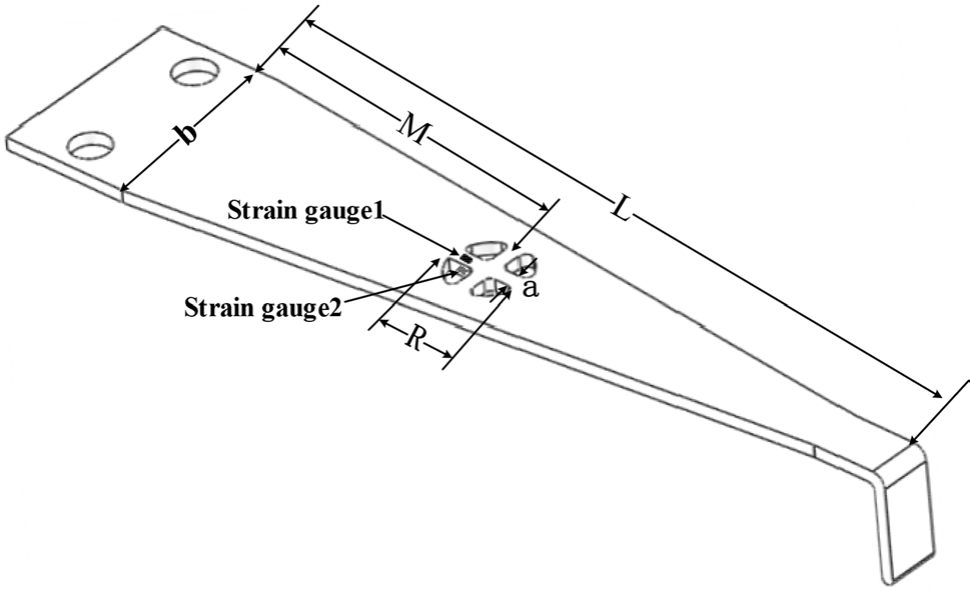
Structural schematic diagram of equal-strength cantilever beam with cross beam (The figure was generated by Microsoft Visio Professional 2016 (https://www.microsoft.com/zh-CN/download/details.aspx?id=51188)).

It can be seen from Fig. 5 that when the displacement measured by the cantilever beam is a fixed value, the larger the $h$ value, the smaller the $L$ value, greater the strain produced on the surface of the cantilever beam, and the higher the sensitivity of the sensor. The traditional method of improving sensitivity generally adopts reducing the length $L$ of the beam, increasing the thickness $h$ of the beam or increasing the height difference of the wedge slider. However, this will inevitably increase the friction between the beam and the slider, the measurement accuracy will be easily impacted by the wear of the beam and slider during long-term reciprocating measurement. Therefore, in this paper, four through-holes were made to the surface of cantilever beam to form a cross beam. The length $L$ and thickness $h$ of the cantilever beam were changed indirectly without changing the size of the original cantilever beam and wedge slider, the surface strain of the cross beam was increased, the sensitivity of the sensor was improved, and the influence of the size increase of the cantilever beam and wedge slider on the long-term reciprocating measurement accuracy of the sensor was avoided. The dimensions of the cross beam are: the distance $M$ from the center of cross beam to the free end of cantilever beam, the width $a$ of cross beam and the diameter $R$ of the through-holes, and its structure is shown in Fig. 7.

**Figure 8:**
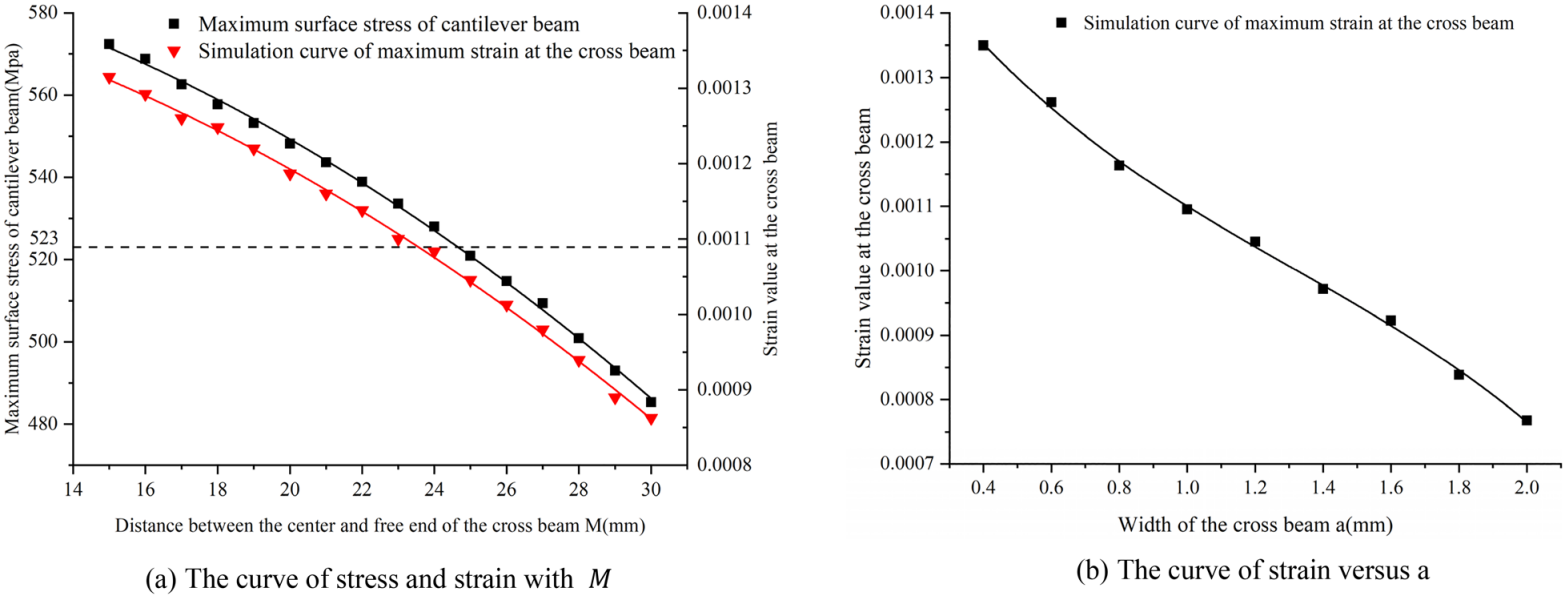
Influence of parameters of the cross beam on the surface strain of the cantilever beam ((**a**,**b**) were generated by Origin 2016 × 64 (https://www.originlab.com/2016)).

When a through-hole were drilled to the surface of the cantilever beam, the strain generated on the surface of the beam concentrated in the hole area. The larger the diameter of the hole, the greater the concentrated strain in this area. The mechanical properties of materials tell people that in the elastic deformation of metal materials, the surface strain of cantilever beam increases at the same time, its surface stress also increases continuously. However, the surface stress must be less than the allowable stress of the material of cantilever beam, otherwise the cantilever beam will be permanently deformed and damaged. Therefore, the positions of the through-hole are generally located between the middle line of the isosceles trapezoid of the beam and the upper bottom, and there should be a safe distance of more than 2 mm between the boundary of the through-hole and the boundary of the cantilever beam. Therefore, to increase the surface strain of cantilever beam within the limited allowable stress range of materials and obtain higher sensitivity, the size of through-holes on the cross beam was selected as R = 6 mm. When other parameters were determined, Solidworks software was used to model cross beams with different $M$ and *a*. Furthermore, based on ANSYS, static simulation analysis was carried out to study the influence of two key parameters $M$ and *a* on the surface strain of cantilever beam, so as to obtain the best parameter of the cross beam. The simulation results and their fitting curves are shown in Fig. 8.

It can be seen from Fig. 8 that when the distance $M$ from the center of the cross beam to the free end of the cantilever beam varies from 15 to 30 mm, the maximum strain on the surface of cross beam and the maximum stress on the surface of cantilever beam decrease with the increase of $M$. The cantilever beam is made of 65Mn spring steel, the allowable stress of which is 523Mpa. In order to obtain the optimal strain within the allowable stress, the distance between the center of the cross beam and the free end of the cantilever beam is selected as $M=25$ mm. When $M=25$ mm, and the width $a$ of the cross beam varies from 0.4 to 2 mm, the maximum surface strain of cross beam also decreases with the increase of $a$. In order to obtain higher sensitivity without impacting the measuring range of the sensor, the distance from the center of the cross beam to the free end of the cantilever beam was selected as $M=25$ mm, the width of the cross beam $a=1.2$ mm, the dimension of the through-hole $R=6$ mm, the length of the cantilever beam $L=60$ mm, the thickness $h=1$ mm and the width $b=15$ mm.

### Design of circuit

**Figure 9:**
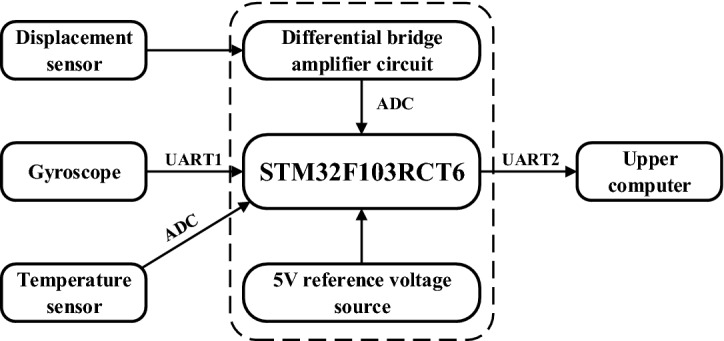
Block diagram of the circuit (The figure was generated by Microsoft Visio Professional 2016 (https://www.microsoft.com/zh-CN/download/details.aspx?id=51188)).

The data acquisition circuit of the sensor is shown in Fig. 9, which is mainly composed of gyroscope, temperature sensor, differential bridge amplifier circuit and single chip microcomputer control module. The temperature sensor collects the ambient temperature of the sensor in real time, and the single chip microcomputer control module uses linear temperature compensation method to eliminate the adverse effects caused by temperature according to the current temperature. The differential bridge amplifying circuit outputs the resistance signal of the strain gage through the bridge and the operational amplifier, and the single chip microcomputer control module collected the temperature data of the temperature sensor, the voltage of the differential bridge amplifier circuit and the angle data of the gyroscope through AD and UART. Through calculation, the 3D displacement data of the seismic isolation bearing was obtained and output to the upper computer for storage.

**Figure 10:**
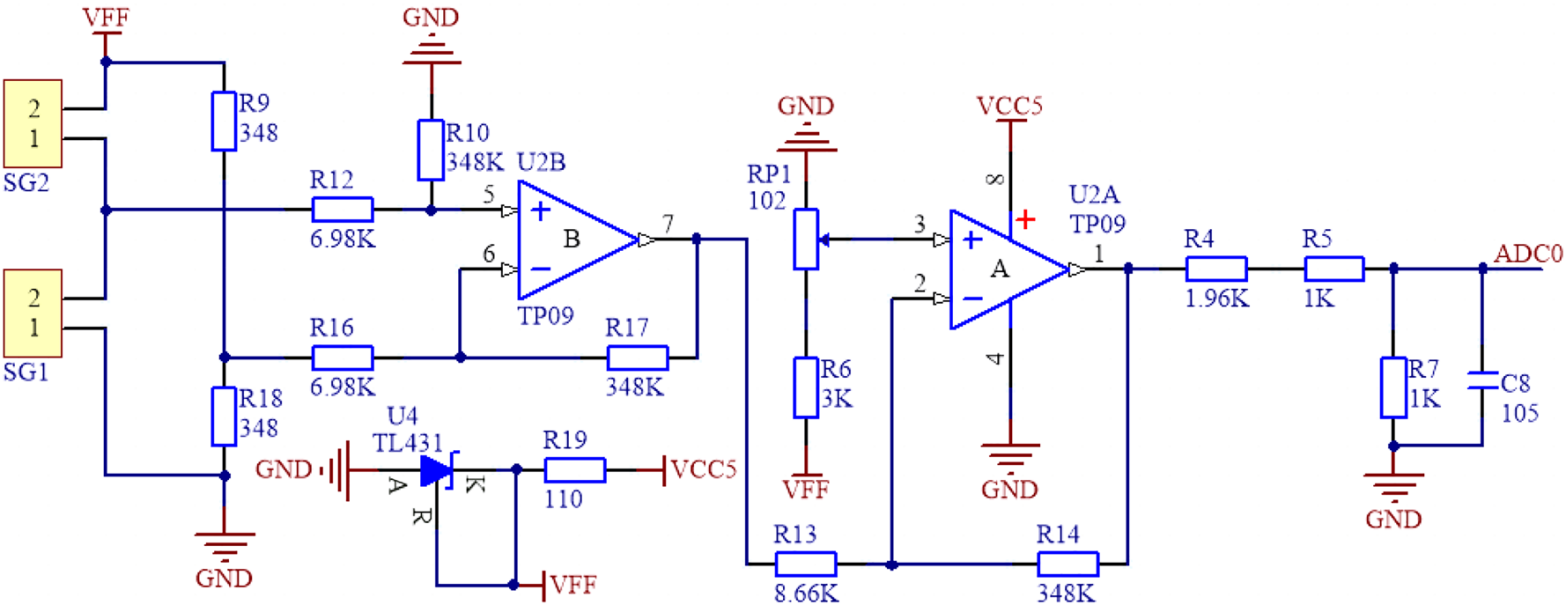
Differential bridge amplifier circuit (The figure was generated by Altium Designer 2016 (https://www.altium.com.cn/products/downloads)).

In the differential bridge amplifier circuit, the two strain gauges ${SG}_{1}$ and ${SG}_{2}$ were identical and in the same temperature environment. When the sensor was subjected to displacement, for the resistance of the strain gauges ${SG}_{1}$ and ${SG}_{2}$, one increased and the other decreased. Since they were under the same temperature, the resistance changes caused by temperature change were the same. When they were connected to two adjacent arms of the bridge, the nonlinear error and temperature error of the strain gauge itself could be compensated, the measurement sensitivity and accuracy could be improved at the same time. Since the output signal of the strain gauge was very weak, in order to facilitate the acquisition and processing of single chip microcomputer control module, this paper has used TP09 operational amplifier to amplify the signal. The component has the advantages of low offset, low power consumption, high precision and high common mode rejection ratio. The weak voltage signal was amplified by 600 times with 0.1% precision resistor, and the voltage signal of 0–5 V was output. The circuit is shown in Fig. 10.

STM32F103RCT6 with ARM core was selected as the main controller of MCU control module, which was integrated with three 12-bit ADCs and two initial serial ports, and can meet the requirements of voltage, temperature and angle signal acquisition. The gyroscope was WT931 attitude inclination sensor module of Witmotion Company. Its measuring range was X, Z ± 180° and Y ± 90°, and its measuring accuracy was 0.05° in X and Y and 1° in Z axis. The gyroscope module communicates with STM32 single chip microcomputer through serial port TTL, which has the advantages of small size, high precision and fast return rate. DS18B20 high-precision digital temperature module was used as the temperature sensor to realize real-time temperature measurement. The temperature module was small in size and highly integrated, and could communicate with single chip microcomputer without other peripheral components.

### Linear temperature compensation model

**Figure 11:**
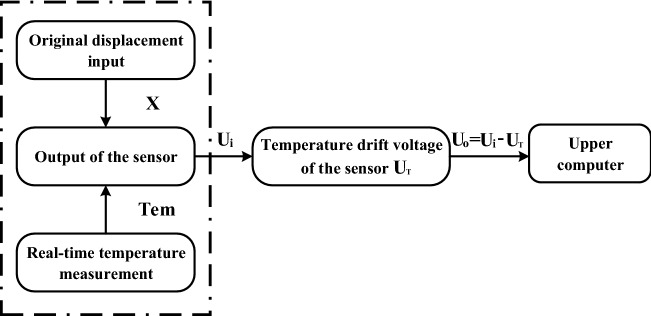
Software linear temperature compensation model (The figure was generated by Microsoft Visio Professional 2016 (https://www.microsoft.com/zh-CN/download/details.aspx?id=51188)).

Although the differential bridge amplifier circuit can reduce part of the error of temperature drift, its compensation accuracy is limited, which cannot meet the high-precision measurement requirements of the sensor. To further reduce the influence of temperature drift on the output voltage of the sensor, the paper has used DS18B20 high-precision temperature sensor to collect temperature in real time, and has constructed a software linear temperature compensation model, as shown in Fig. 11.

Under the influence of ambient temperature change, the output voltage ${U}_{i}$ of the sensor is not only a function of the applied displacement $X$, but also a function of the temperature $T$. When the standard displacement $X_{i}$ is applied to the sensor, its indication value is:

18 $$\begin{array}{*{20}c} {U_{i} = f\left( {X_{i} ,T} \right)} \\ \end{array}$$

where $U_{i}$ is the voltage value displayed by the sensor before temperature compensation under the application of standard displacement X.

Under the room temperature of 23 °C, n detection points were selected for displacement calibration of the displacement sensor used in the experiment, and the output voltage of the sensor is as follows:

19 $$\begin{array}{*{20}c} {U|_{T = 23} = f\left( {23} \right)} \\ \end{array}$$

Change the temperature of the temperature control box without applying any displacement action to the sensor, the output voltage of the displacement sensor at different temperatures were measured as follows:

20 $$\begin{array}{*{20}c} {U = f\left( T \right)} \\ \end{array}$$

By using the linear fitting algorithm based on the least square method, the temperature calibration equation was obtained by linear fitting of the indication value of the sensor at different temperatures:

21 $$\begin{array}{*{20}c} {U_{T} = f\left( T \right) = a*T + b} \\ \end{array}$$

where $a$ and $b$ are the linear fitting coefficients, $T$ is the real-time temperature, and $U_{T}$ is the temperature drift voltage at the current time.

At last, the difference between the measured voltage $U_{i}$ at the current moment and the temperature drift output voltage $U_{T}$ under the current temperature condition were made by the program of single chip microcomputer, and the compensated output voltage $U_{o}$ is obtained:

22 $$\begin{array}{*{20}c} {U_{o} = U_{i} - U_{T} } \\ \end{array}$$

## Performance test and analysis

### Temperature compensation experiment

**Figure 12:**
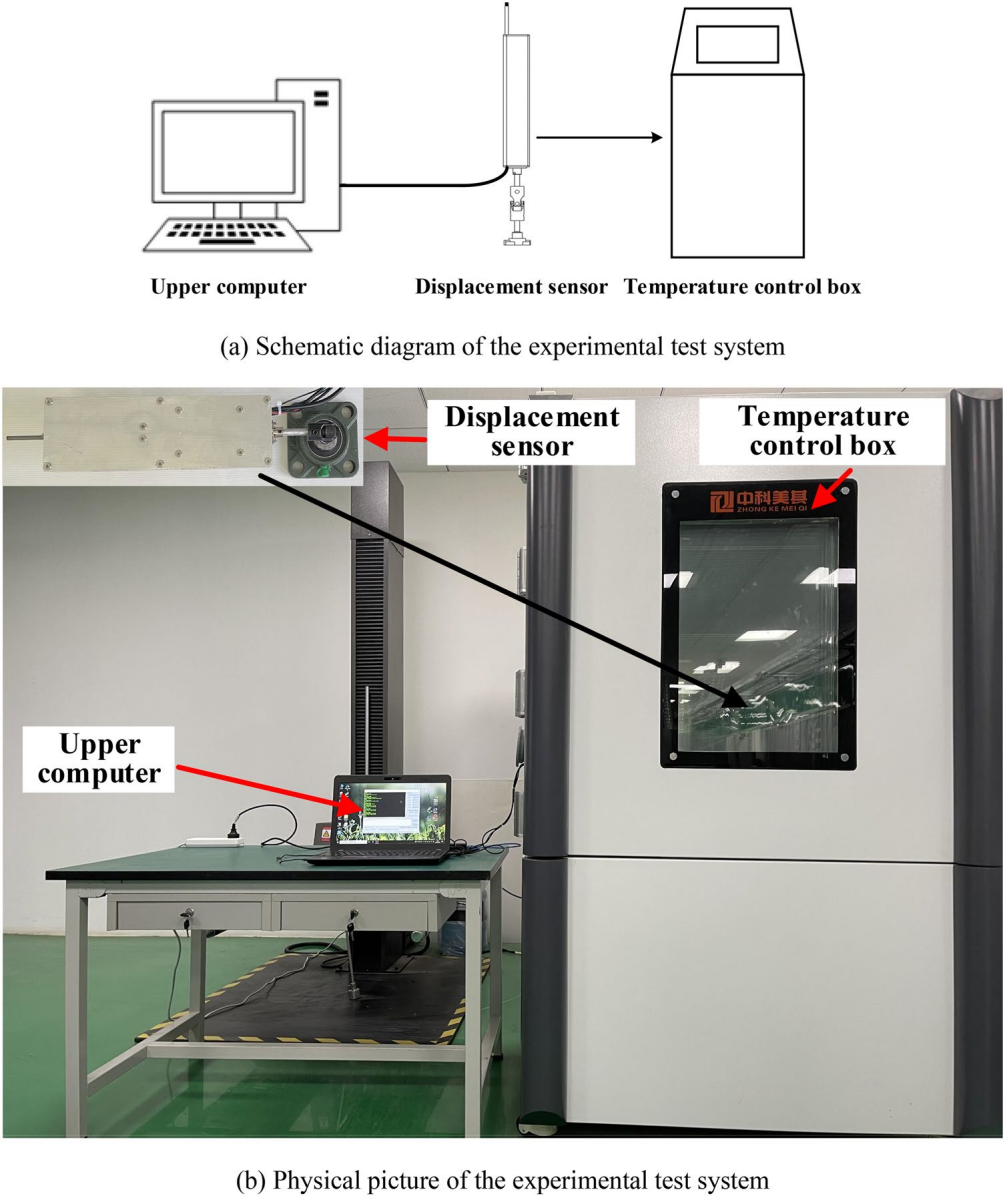
Schematic diagram of the temperature experimental test system ((**a**,**b**)were generated by Microsoft Visio Professional 2016 (https://www.microsoft.com/zh-CN/download/details.aspx?id=51188)).

According to the size of the displacement sensor, the high-sensitivity rotatable 3D displacement sensor is shown in Fig. 12b. In order to study the temperature performance of the 3D displacement sensor, a temperature compensation test system for the displacement sensor was built, as shown in Fig. 12. The sensor was put into the MQ-TH1000F-2N temperature control box produced by Tianjin Zhongke Meiqi Technology Co., Ltd. (measuring range: − 70 to 170 °C, accuracy: 0.01 °C), and the output voltage of the sensor was collected by the upper computer in real time.

**Figure 13:**
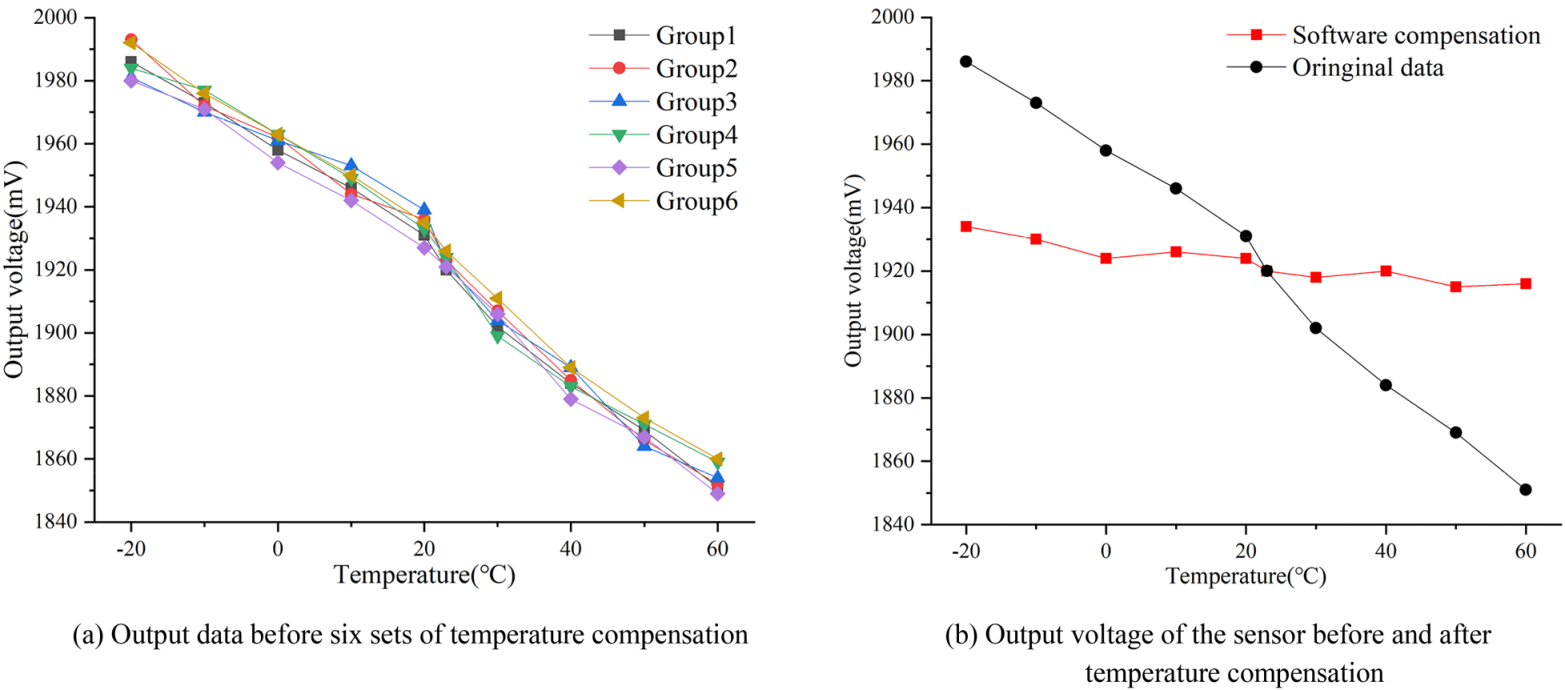
Comparison of the output voltage of the sensor before and after temperature compensation ((**a**,**b**) were generated by Origin 2016 × 64 (https://www.originlab.com/2016)).

Firstly, in order to determine the fitting coefficient of the temperature calibration equation, the temperature of the temperature control box was raised from − 20 to 60 °C. Every 10 °C was a gradient, and each time the temperature was maintained for 15 min. After the temperature indication value in the box was stable, the temperature displayed by the temperature control box and the output voltage drift of the sensor were recorded. Six groups of data were measured repeatedly, and the curve of output changing with temperature is shown in Fig. 13a. Since the temperature drift data was fluctuating, the average value of six groups of measured data was taken and the temperature drift data of the sensor was linearly fitted by the least square method, and the output voltage equations of temperature drift at different temperatures were obtained. The fitting equation is as follows:

23 $$\begin{array}{*{20}c} {U_{T} = - 1.73*T + 1957.29} \\ \end{array}$$

The temperature calibration equation was input into the MCU program, correcting the output voltage of the sensor, to realize temperature compensation. By repeating the above experimental flow, the output voltage curves before and after temperature compensation were obtained, as shown in Fig. 13b.

As can be seen from Fig. 13b, after temperature compensation, the output voltage of the displacement sensor slowed down with temperature, and the output range error decreased from 135 to 19 mV. The temperature drift was improved to some extent. The maximum temperature drift error was reduced from 3.6 to 0.7%, indicating that the temperature compensation model of the sensor was rational and could be used for effective temperature compensation.

**Figure 14:**
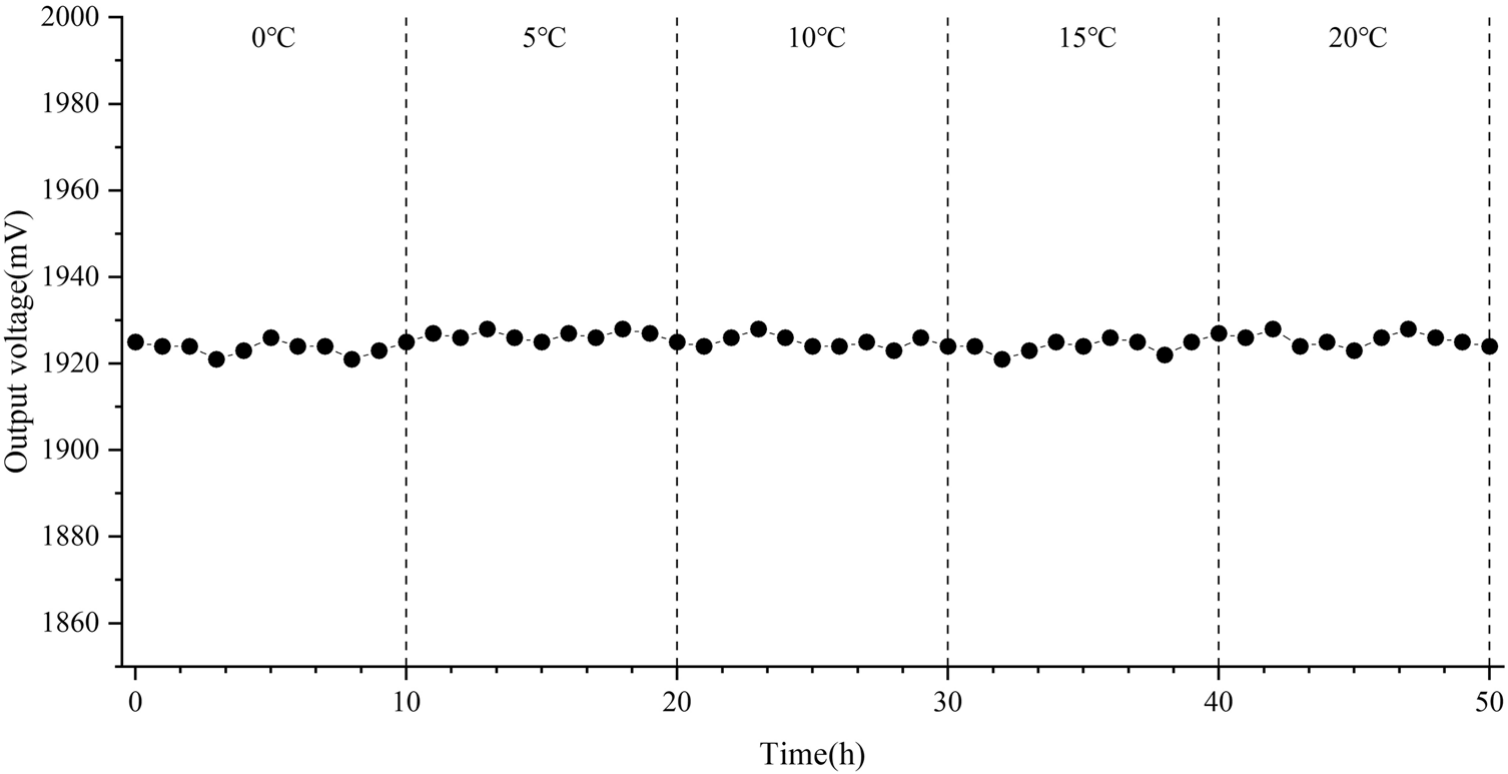
Sensor output voltage curve in different temperature ranges (The figure was generated by Origin 2016 × 64 (https://www.originlab.com/2016)).

Secondly, since the ambient temperature of the isolation layer where the isolation bearing is located is between 0 and 20 °C all the year round, in order to further verify the long-term temperature stability of the sensor within this range, the temperature of the temperature control box was raised from 0 to 20 °C, and every 5 °C is a gradient, and each time is kept for 10 h. After the temperature indication in the box is stable, the voltage value is recorded every 1 h. The duration of the experiment last about 50 h. The output voltage curve of the sensor is shown in Fig. 14.

As can be seen from Fig. 14, the output voltage of the sensor fluctuates gently within the temperature range of 0–20 °C. Taking the sampling value of the temperature point at 23 °C as the reference point, the maximum temperature drift error is only 0.4%, which indicates that the sensor has good stability under long-term constant temperature.

### Static calibration experiment

**Figure 15:**
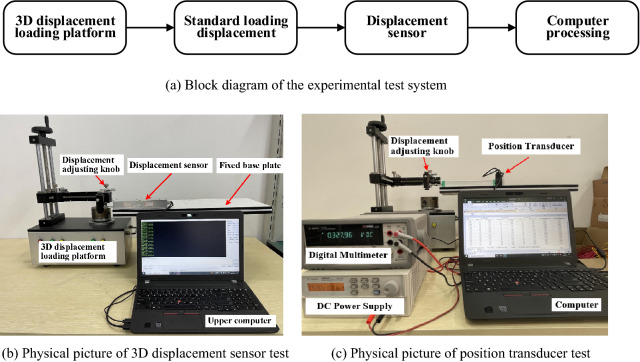
Static calibration system of the displacement sensor ((**a**,**b**,**c**)were generated by Microsoft Visio Professional 2016 (https://www.microsoft.com/zh-CN/download/details.aspx?id=51188)).

To compare the static performance of different displacement sensors, the comparison experiment is conducted by using the KTC-160 mm position transducer produced by Hermitt. Then, a static calibration system for the displacement sensor is built which has a measuring range of – 900 to 900 mm and an accuracy of 0.5 mm, by using the TD8411 rotating platform surface magnetic distribution tester produced by Tianheng Measurement and Control Company, along with the 34410A digital multimeter and the U8002A DC power supply produced by Agilent Company. The system block diagram and physical diagram are shown in Fig. 15, respectively.

**Figure 16:**
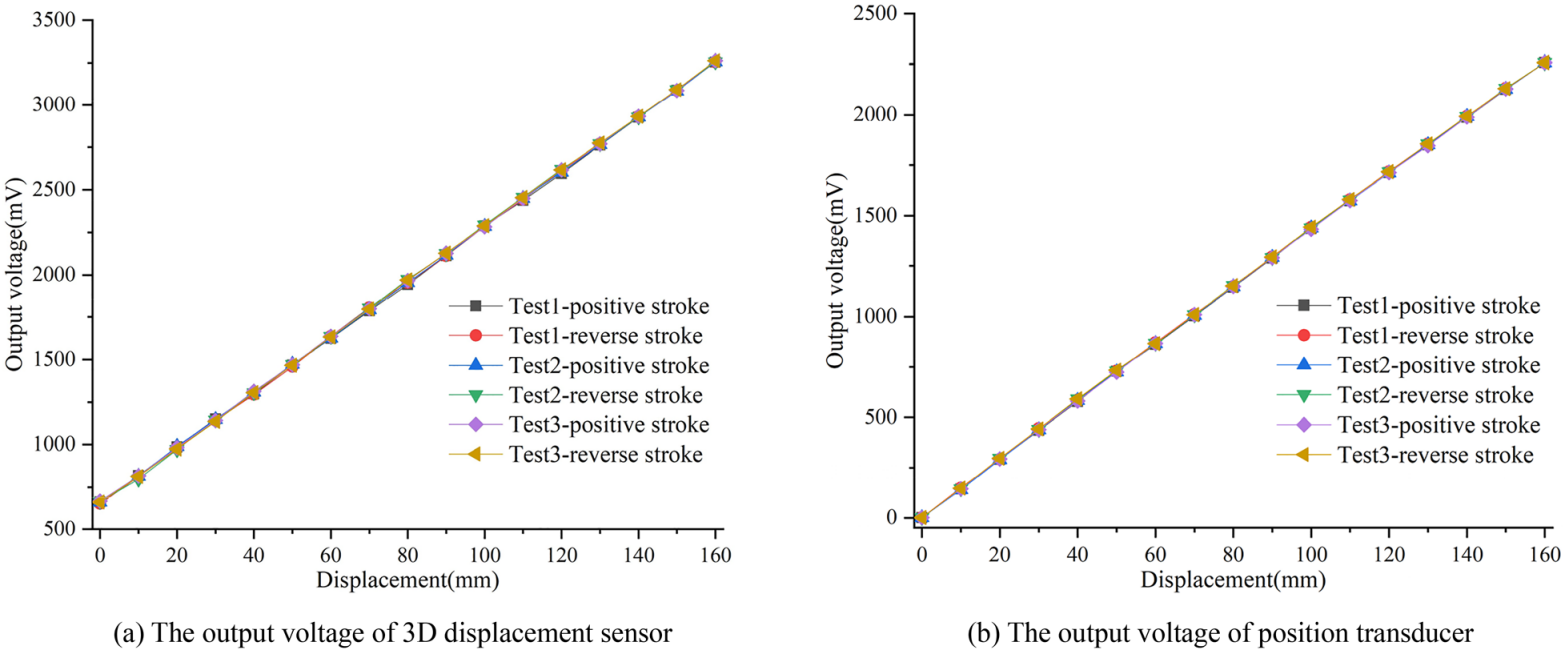
Time history diagram of 3 displacement tests ((**a**,**b**) were generated by Origin 2016 × 64 (https://www.originlab.com/2016)).

Fix the clamping end of the 3D displacement loading platform with the measuring guide rod of both the 3D displacement sensor and position transducer, then rotate the displacement adjustment knob in the X direction with a step size of 10 mm, and pull the scale of 0 mm to full 160 mm in sequence according to the positive stroke; Stop at each displacement point for 3–5 s until the output voltage is stable, then record the voltage data at this time; After recorded, reduce the displacement from 160 to 0 mm in reverse stroke. The whole process should be continuously tested for 3 times, Fig. 16 shows the output voltage value corresponding to each displacement point in the 3 times experiments.

**Figure 17:**
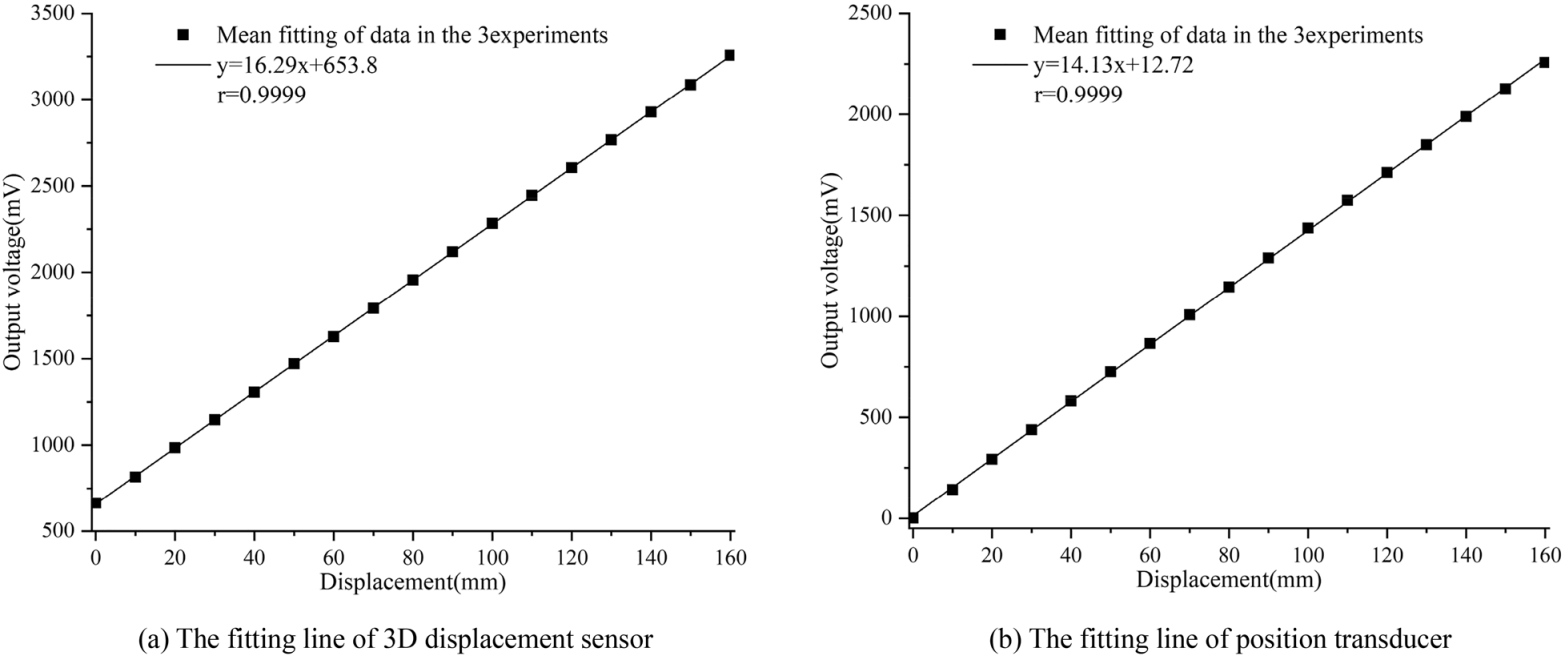
Linear fitting line of output voltage value and displacement ((**a**,**b**) were generated by Origin 2016 × 64 (https://www.originlab.com/2016)).

The arithmetic mean value of the six groups of data in Fig. 16 was obtained, and the data were fitted in a straight line by the least square method. The results are shown in Fig. 17.

It can be seen from the fitting line that when the measuring range is 160 mm, the displacement sensitivity of the 3D displacement sensor is 16.29 mV/mm, the linear correlation coefficient is 0.9999, the linearity is 0.36%, the hysteresis error is 0.45%, and the repeatability error is 0.69%. The total accuracy of the sensor can be calculated to be 0.9% by square sum root method. In comparison, the position transducer has a displacement sensitivity of 14.13 mV/mm, a linear correlation coefficient of 0.9999, a linearity of 0.8%, a hysteresis error of 0.37%, and a repeatability error of 0.27%, with a total accuracy of 0.92%.

In addition, the test results show that the 3D displacement sensor has a 15% higher sensitivity compared to the commercial position transducer. While the linearity, hysteresis error and repeatability error of the two displacement sensors have their own advantages and disadvantages, the total accuracy is comparable. Therefore, the 3D displacement sensor shows better displacement measurement performance.

### Static 3D space displacement experiment

**Figure 18:**
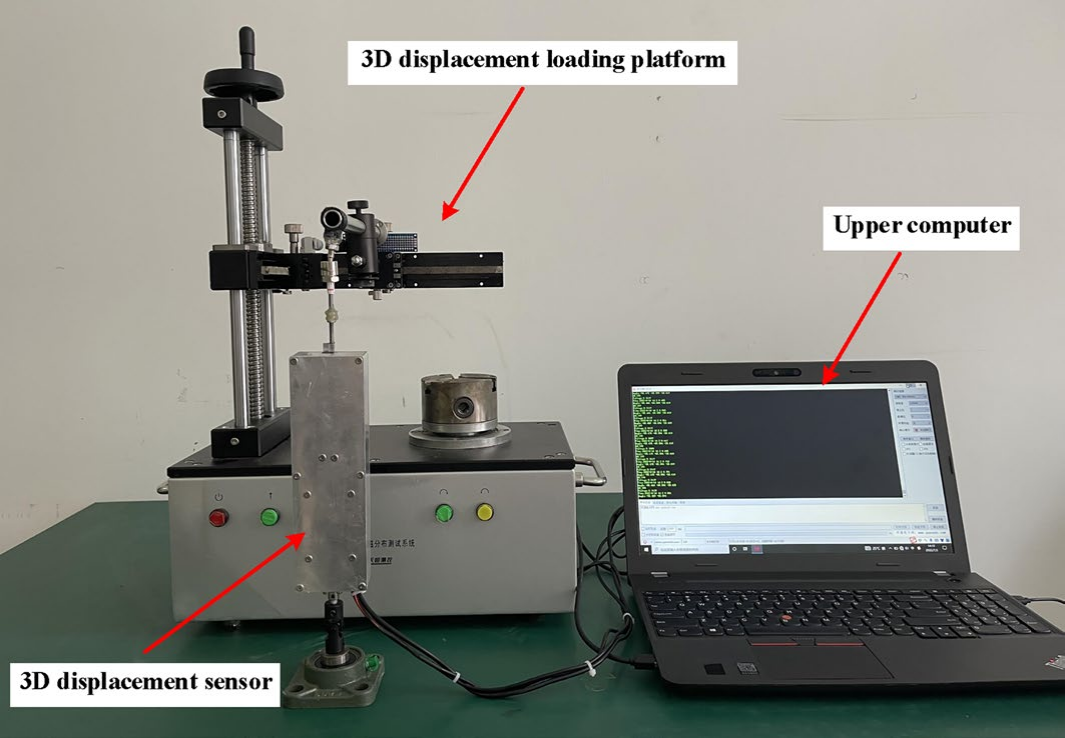
3D space displacement test platform (The figure was generated by Microsoft Visio Professional 2016 (https://www.microsoft.com/zh-CN/download/details.aspx?id=51188)).

In order to further verify the accuracy and reliability of the sensor's static 3D displacement measurement, a 3D displacement test platform was rebuilt using the TD8411 rotating platform surface magnetic distribution tester, as shown in Fig. 18. Fixed the measuring guide rod of 3D displacement sensor on the 3D displacement loading platform, using the displacement knob on the platform to move the test point at a fixed distance and direction in space, thereby driving the 3D displacement sensor to measure the guide rod to move at a fixed distance and direction. The output voltage and angle of the sensor were recorded by the upper computer, and the 3D stretching displacement of the sensor was obtained.

**Figure 19:**
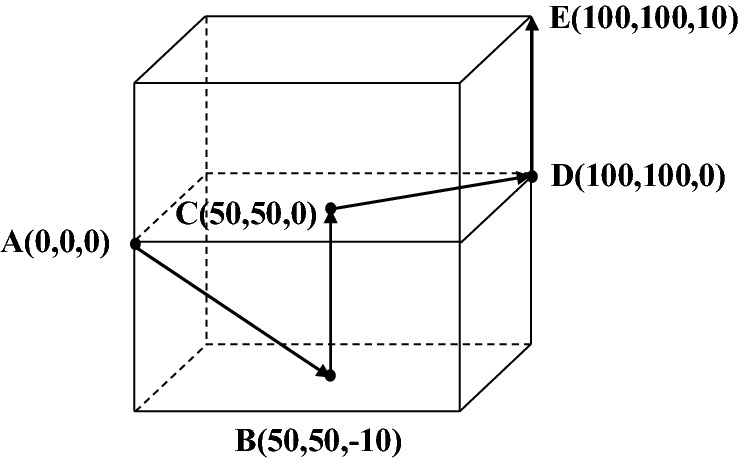
Schematic diagram of the movement of measuring points (The figure was generated by Microsoft Visio Professional 2016 (https://www.microsoft.com/zh-CN/download/details.aspx?id=51188)).

The initial fixed position of the 3D displacement sensor was set as the origin A (0, 0, 0), B (50, 50, − 10), C (50, 50, 0), D (100, 100, 0) and E (100, 100, 10) were preset in the space. Move from point A to point E in turn at a fixed distance and direction, and stay at each point for about 3 s. The schematic diagram of measuring point movement is shown in Fig. 19.

**Figure 20:**
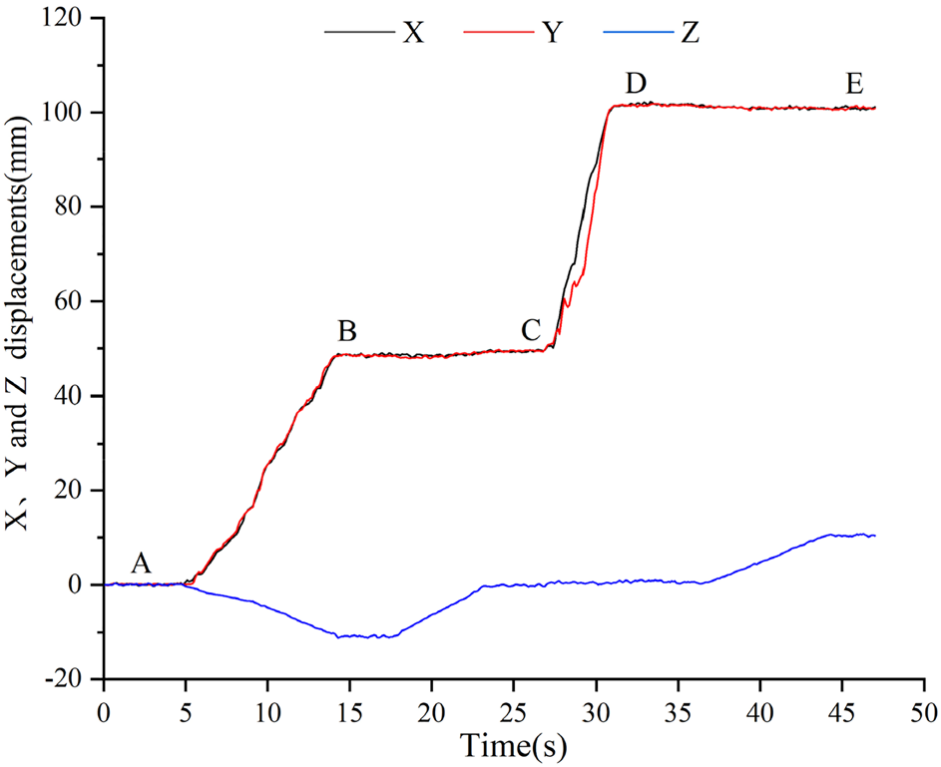
Variation curve of displacements in X, Y and Z directions (The figure was generated by Origin 2016 × 64 (https://www.originlab.com/2016)).

The values of voltage and angle which collected and recorded by the upper computer were calculated by formula, and the corresponding displacement in X, Y and Z directions were obtained. Then, the data waveform was smoothed by Origin, and the 3D displacement waveform curve was obtained as shown in Fig. 20.

As can be seen from Fig. 20, when the displacement stage changes, it will rise or fall with fluctuations, because the artificial rotation of the displacement adjustment knob cannot maintain a constant speed during the stage change. Therefore, the waveform will change according to the rotation amplitude and frequency of the displacement adjustment knob, resulting in irregular waveform changes, which also reflects the change of the waveform with the actual position. When the Z-direction displacement of B–C section decreases and the Z-direction displacement of D–E section increases, the X-direction displacement and Y-direction displacement also increases and decreases in a small amplitude; In the process of increasing the displacement in the X and Y directions of the C–D section, the displacement in the Z direction also increases slightly, because the connection between the sensor measuring guide rod and the clamping end of the 3D displacement loading platform is unstable. When it works, the fixed part of the measuring guide rod and the bearing base will be slightly deformed by the force, which will bring errors to the measurement of the remaining component displacements.

**Figure 21:**
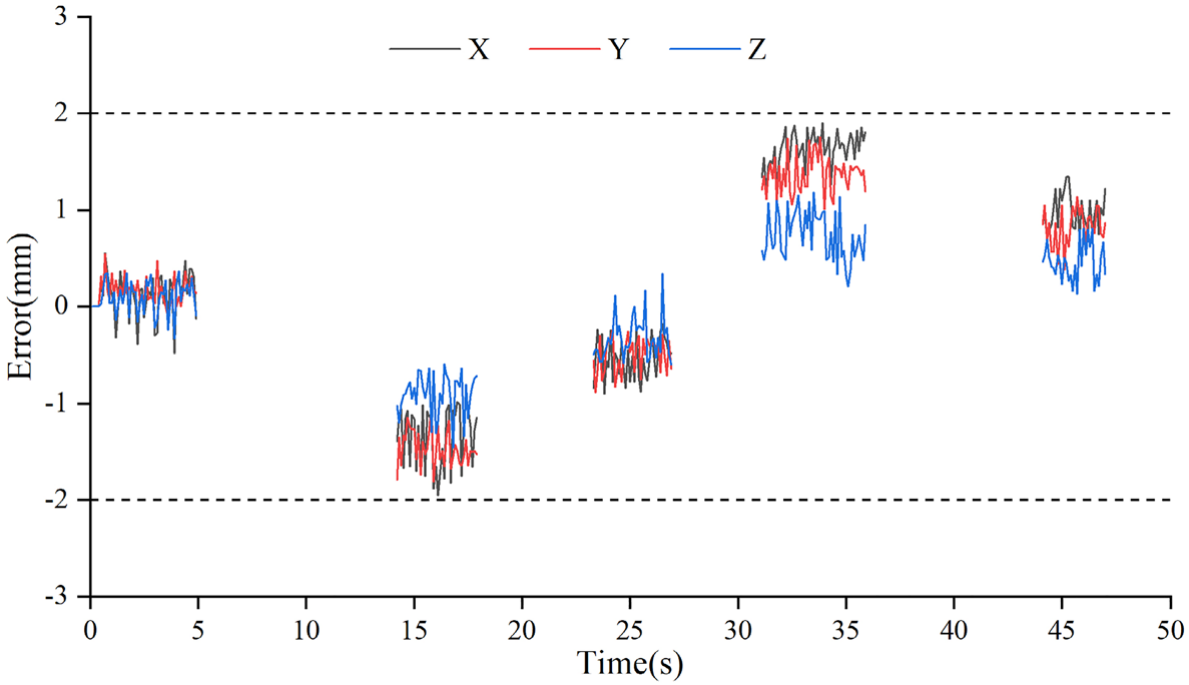
Measured displacement errors in X, Y, Z directions (The figure was generated by Origin 2016 × 64 (https://www.originlab.com/2016)).

The data were intercepted when the sensor was stretched to points A, B, C, D with a stable status, and the coordinates corresponding to the points were subtracted to obtain the displacement measurement errors in X, Y and Z directions, as shown in Fig. 21.

As can be seen from Fig. 21, the measurement errors of displacement data in X, Y and Z directions are all less than 2 mm. The measurement error might be caused by inadequate fixation between the measuring guide rod and the clamping end of the displacement loading platform. When the displacement knob was adjusted manually, the deformation between the displacement loading platform and the sensor occurred, which led to slight errors. The experimental results have shown that the sensor was accurate and reliable in measuring static 3D displacement.

### Displacement experiment in dynamic 3D space

**Figure 22:**
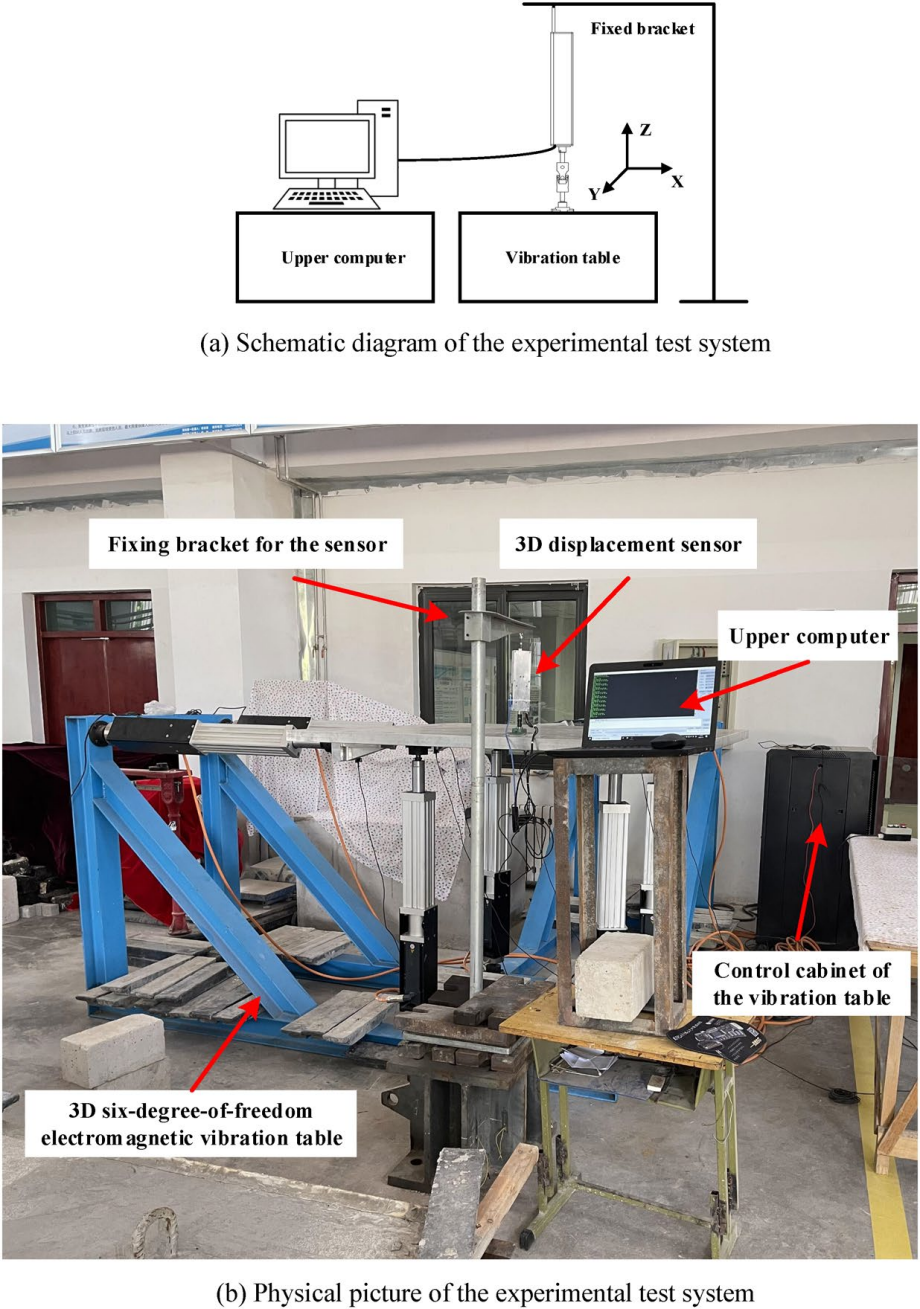
Dynamic 3D space displacement test system ((**a**,**b**)were generated by Microsoft Visio Professional 2016 (https://www.microsoft.com/zh-CN/download/details.aspx?id=51188)).

When the seismic isolated building is subjected to earth vibration, slow translation will happen between the seismic isolation bearings and their connected upper building, and the basic horizontal natural frequency ranges from 0.2 to 0.5 Hz. To verify the dynamic 3D displacement measurement performance of the 3D displacement sensor near the frequency range, a 3D six-degree-of-freedom electromagnetic vibration table of Hebei Provincial Key Laboratory of Earthquake Disaster Prevention and Risk Assessment, Disaster Prevention Technology College, was used to simulate the dynamic displacement of the seismic isolation bearings. The dynamic 3D space displacement test system is shown in Fig. 22. The 3D six-degree-of-freedom electromagnetic vibration table was mainly composed of a table top, a base, a servo electric cylinder and a control cabinet. The size of the table top was 1.5 × 1.5 m, and the maximum bearing weight was 2000 kg. The maximum horizontal acceleration of the table was ± 2 g, the maximum vertical acceleration was ± 1.5 g, the maximum stroke was ± 10 cm, and the working frequency was 0–60 Hz, which could meet the displacement and frequency range required by this experiment.

**Figure 23:**
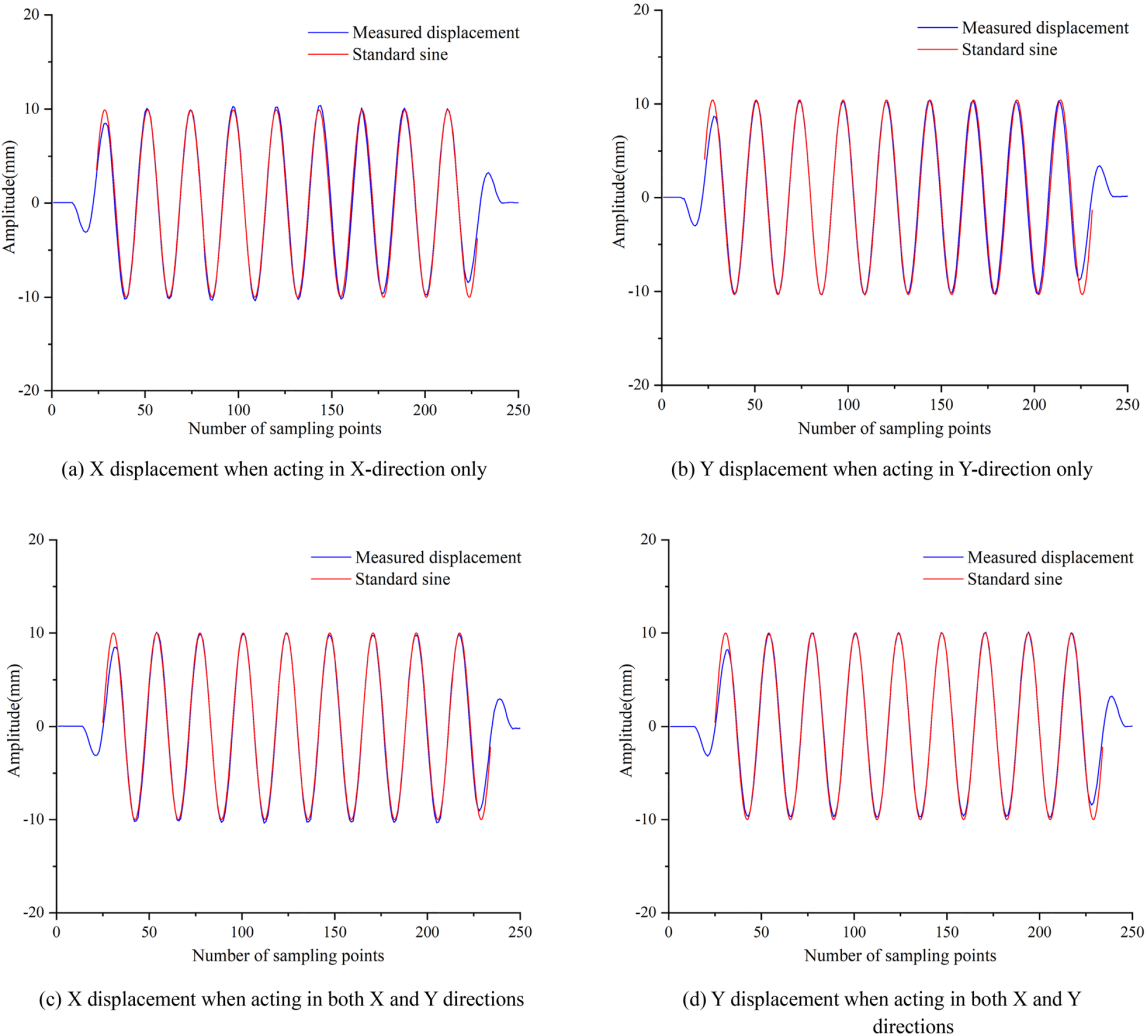
Comparison of measured displacement and standard displacement with measured frequency of 0.9 Hz and displacement of 10 mm ((**a**,**b**,**c**,**d**) were generated by Origin 2016 × 64 (https://www.originlab.com/2016)).

**Figure 24:**
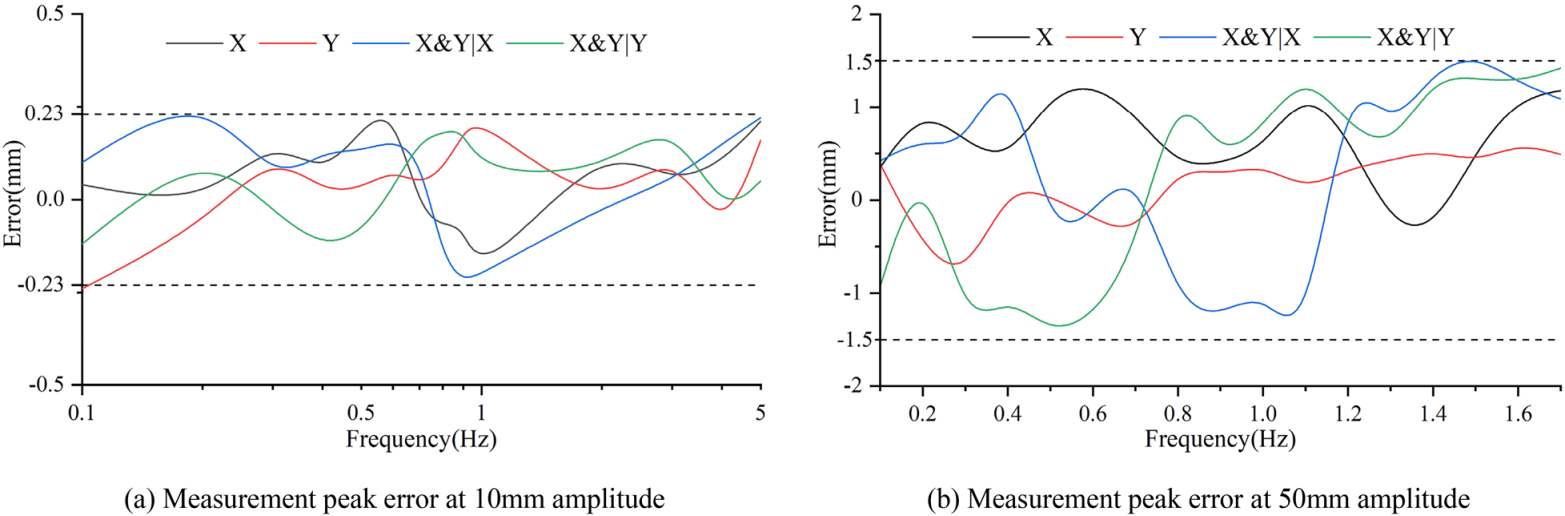
Peak measurement error of the sensor at various frequencies ((**a**,**b**) were generated by Origin 2016 × 64 (https://www.originlab.com/2016)).

Before the experiment, the vibration table was hoisted in a fixed area and a lead counterweight was installed to it to ensure that it can keep running smoothly. The height of the sensor fixing bracket was adjusted, and the displacement sensor measuring guide rod between the bracket and the table top was fixed. The circuit of the sensor and vibration table was checked to eliminate the influence of human error and other factors on the normal work of the system. The experiment process was as follows: adjusting the upper computer of the control end of the vibration table to make the table output sinusoidal excitation signals with displacement amplitude of 10 mm when in X and Y direction alone and X and Y directions together. The frequency range was 0.1–5 Hz. Among them, 0.1 Hz was taken as the step size between 0.1 and 1 Hz, and 1 Hz was taken as the step size between 1 and 5 Hz. Then, the sine displacement amplitude was set as 50 mm, the frequency range was 0.1–1.7 Hz, and the experiment was repeated with 0.1 Hz as the step size. Figure 23 is a comparison curve between displacement data of displacement sensor and standard sine curve at the frequency of 0.9 Hz and displacement amplitude of 10 mm. The maximum peak value of the output displacement of the displacement sensor in each frequency range was selected to make a difference with the measured value, and the measurement error at each frequency test point when the amplitude of the displacement sensor was 10 mm and 50 mm was obtained, as shown in Fig. 24.

As can be seen from Figs. 23 and 24, due to the performance limitation of the vibration table, the displacement amplitude in the initial and final stages could not reach the set target, and the sinusoidal displacement data measured by the sensor in other displacement stages were basically consistent with the standard sinusoidal wave. When the amplitude of sinusoidal displacement was 10 mm, with the change of frequency, the measurement error caused by the single action and the joint action of X and Y directions had a small change range, which was between ± 0.23 mm. The relative error of was 2.5%. However, when the amplitude of sinusoidal displacement was 50 mm, the measurement error caused by the single action and the joint action of X and Y directions varied greatly, both of which were between 1.5 mm. The relative error was 3%, which was smaller than that of 10 mm displacement amplitude measurement, indicating that the sensor has good dynamic displacement measurement performance.

## Conclusion

In this paper, a high-sensitivity rotatable 3D displacement sensor was proposed. The cantilever beam and equal-strength mechanical structure of the sensor were specially designed to improve the sensitivity and accuracy of the sensor and realize 3D displacement measurement at the same time. Through the combination of simulation analysis with experimental verification, the optimal design and performance test of the designed sensor were carried out. The results have shown that the measuring range of the sensor is 160 mm, the sensitivity is about 16.29 mV/mm, the accuracy can reach 0.9%, and the static and dynamic 3D displacement measurement errors are less than 2 mm. Compared with other types of displacement sensors used in the health monitoring of seismic isolation bearings, the equal-strength cantilever beam of the sensor designed in this paper adopts a cross beam type through-hole design, which increases the bending strain of the beam surface to improve the sensitivity; After adding a gyroscope and mechanical rotating structures, a sensor can be used to measure the three-dimensional displacement of the isolation bearing, at the same time it can be used to reduce the adverse effect of the displacement transmission mechanism on the measurement accuracy. It has the advantages of high sensitivity, high measurement accuracy, and strong applicability. However, there leaves much space to improve to some extent. In fact, the working environment of the isolation bearing is generally dark and humid, if the sensor works on this state for a long time, the performance of the sensor will be affected, because the internal mechanical and electronic components of the sensor may fail in this condition. Therefore, the original scheme can be further improved, so that the sensor can be applied to the health monitoring study of seismic isolation bearings in bridges, high-rise buildings, water conservancy and other engineering fields as soon as possible.

## Data Availability

The datasets used and/or analyzed during the current study are available from the corresponding author upon reasonable request.
